# Supplementary material for: Factors That May Affect Breast Milk Macronutrient and Energy Content: A Critical Review
Source: Nutrients. 2025 Jul 30;17(15):2503. doi: 10.3390/nu17152503 (PMC12348597; doi:10.3390/nu17152503)
Supplement: Supplementary file 1 [file nutrients-17-02503-s001.zip › nutrients-3741127-supplementary.pdf]

nutrients MDPI

Supplementary Table S1. Studies on the effects of maternal, obstetrical, and neonatal factors on the macronutrient and energy content of breast milk, published between 2006-2025, presented in decreasing chronological order.

Reference	Study design, period of study	Sample size	Factors analyzed	Human milk analyzer	Outcomes	Level of evidence of original studies	Limitations
Hashemi Javaheri, et al. (2025) [9]	Systematic review of 83 studies	11,310 mothers	Maternal BMI	Not specified	Maternal BMI was associated with a higher ratio of n-6/ n-3 PUFAs in breast milk<br>No conclusive associations were found between maternal BMI and milk energy and macronutrients content	Not applicable	Not acknowledged
Arenas, et al. (2025) [14]	Narrative review	Not specified	Obesity<br>Diabetes<br>Hypertension<br>Dyslipidemia<br>Metabolic syndrome	Not specified	The milk from obese mothers had a higher total fat and carbohydrates content and an increased n-6/ n-3 PUFAs ratio<br>The colostrum from mothers with GDM had a higher carbohydrates and energy content<br>The milk from mothers with pregestational diabetes mellitus had a higher glucose content, and a lower long-chain PUFAs content<br>The milk from mothers with hypertension had a higher total protein, fat, carbohydrate and energy content compared with normotensive mothers<br>The milk from mothers who had gestational hypertension had a lower fat and energy content than the milk from normotensive mothers<br>The milk from mothers with preeclampsia had a lower DHA content	Not applicable	Not acknowledged
Petersohn, et al. (2024) [5]	Systematic review of 27 studies	7138 mothers	Maternal dietary intake	Not specified	Maternal fish intake was positively associated with the milk ALA ($r = 0.28–0.42$), DHA ($r = 0.24–0.46$), and EPA ($r = 0.25–0.28$) content<br>Dietary PUFAs was positively correlated with their content in milk<br>Saturated fatty acids intake was negatively associated with several fatty acids in the milk	Not applicable	Included malnourished populations and supplementation studies

							Diverse methodology, including various dietary assessment methods<br>Other factors associated with milk composition other than the dietary intake
Favara, et al. (2024) [8]	Systematic review of 20 studies, 5 assessing as factors the maternal dietary intake and/or nutritional status, and 3 smoking habits	Not specified	Maternal dietary intake<br>Maternal nutritional status	Not specified	A positive association was found between the maternal protein intake and the protein, fat, carbohydrates, and energy content in milk<br>A negative association was found between maternal fat intake and the EPA and DHA content in milk<br>Overweight or obese mother conditions positively associated with saturated fatty acids, n-6/n-3 ratio, and monounsaturated fatty acids content, and negatively associated with ARA, total n-3 fatty acids, ALA, and DHA content, compared with normal-weight mothers	Not applicable	Heterogeneity across the studies (sample sizes, methodologies, and geographical locations)<br>Methodological inconsistencies<br>Predominant reliance on observational data
Chathyushya, et al. (2023) [35]	Cross-sectional	120 mothers of term and preterm infants	Socioeconomic status	Kjehdahl method for protein, chloroform for fat, carbohydrate	The milk from mothers with a lower socioeconomic status had significantly higher levels of monounsaturated and n-9 fatty acids, while mothers with an upper socioeconomic status had significantly higher levels of n-3 and n-6 polyunsaturated	II-1	Not acknowledged

				and energy estimated			
Correia, et al. (2023) [13]	Historical cohort, during the first 4 weeks postpartum	73 mothers delivering before 37 weeks of gestation	Gestational age<br>Multiple pregnancy<br>Hypertensive pregnancy disorders<br>IUGR	Miris†	Giving birth before 28 weeks of gestation was associated with a higher total energy and true protein milk content<br>Single pregnancy was weakly associated with a higher total energy content in the milk<br>Chronic hypertension was associated with a higher milk energy content<br>Chronic hypertension and hypertension induced by pregnancy were associated with a higher true protein content in the milk<br>The milk fat content was weakly and negatively associated with IUGR, both in SGA infants and AGA infants with fetal growth deceleration (average 0.44g/dL; 95% CI: 0.92 to 0.05; p=0.079 and average 0.36g/dL; 95% CI: 0.74 to 0.02; p=0.066, respectively)	II-1	Convenience sampling<br>IUGR diagnosed by attending obstetricians, with no measure of interobserver variation<br>Accuracy of the diagnosis of IUGR could not be confirmed by Doppler flow profiles<br>Variability in milk energy and macronutrients content dependent on duration of lactation was not considered
Marano, et al (2023) [44]	Cross-sectional	181 mothers	Pre-pregnancy obesity and gestational weight gain<br>Maternal habits, comorbidities,	Miris†	The milk from mothers with pre-gestational obesity and gestational weight gain above the recommendation had a lower protein content compared to eutrophic mothers (median=0.8, interquartile range (IQR): 0.7–0.9 vs. median=0.8, and IQR: 0.8–1.0) and those with adequate gestational weight gain (median=0.8, IQR: 0.7–0.9 vs. median=0.9, and IQR: 0.8–1.0), respectively	II-1	Non-randomized sampling<br>Small number of women with characteristics of interest and a

			and perinatal factors				large number of white women with a university degree
Khelouf, et al. (2023) [58]	Prospective, during 720 days postpartum	92 mothers	Sexual dimorphism	Biuret method for protein; Formula for calculation of carbohydrate; Modified Folch extraction procedure for fat; standard formulas for estimating energy	The colostrum and mature milk from mothers of males had a lower carbohydrate and lactose content, while their mature milk had a higher fat content	II-1	Not acknowledged
Borràs-Novell, et al. (2023) [7]	Prospective, during the first 8 weeks postpartum	117 mothers delivering before 32 weeks of gestation	Gestational age<br>Maternal age<br>Maternal BMI<br>Multiple pregnancy<br>Hypertensive pregnancy disorders<br>IUGR<br>Preeclampsia<br>Labor	Miris†	Gestational age was negatively correlated with total energy (r: −0.229, p = 0.018), fat (r: −0.202, p=0.038) and true protein (rho: −0.337, p<0.001) content in milk<br>The milk from mothers 35 years old or older had a higher protein content, compared with the milk from younger mothers<br>Maternal age was weakly positively correlated with protein content in the 1st (r =0.216, p=0.037) and 2nd week postpartum (r=0.322, p=0.001)<br>The milk from mothers of singleton IUGR infants had a higher protein content at or after 4 weeks postpartum than the milk from mothers of singletons with normal growth	II-1	The macronutrient and energy content was not differentiated between colostrum and transitional milk.

			Lactation history		Advanced maternal age, gestational age, and IUGR were independently associated with milk protein content during the first 4 weeks postpartum (adjusted R^2: 0.113, p=0.002)<br>Overweight and obese mothers had a higher protein, fat, and energy content in mature milk during the first 4 weeks postpartum<br>The milk from mothers delivering multiples was associated with a lower total protein and energy content during the first 4 weeks postpartum, compared with milk from mothers delivering singletons<br>The milk from mothers with hypertensive disorders during pregnancy was associated with a lower fat and energy content in early milk, compared with the milk from normotensive mothers<br>Early milk from mothers with preeclampsia was associated with a lower fat and higher protein content<br>The milk from mothers who had been in labor was associated with a lower protein content at 4 weeks postpartum, compared with the milk from mothers without labor<br>The milk from mothers who previously breastfed, for longer, was positively moderately correlated with protein content during the first 4 weeks postpartum (rho: 0.436, p =0.029).		
Bottin, et al. (2022) [36]	Prospective, at weeks 1, 4, 11, 18, and 25 postpartum	48 mothers	Socioeconomic status	Various	High food insecurity indexes were significantly associated with lower fatty acid (aß-coef =–7.2, p value = 0.03), retinol (aß-coef =–0.2, p value = 0.04), and amino acids (aß-coef =–2121.0, (p < 0.001) and higher lactose content in the breast milk.<br>Intake of meat, poultry, and fish was associated with higher total amino acid content and lower lactose content (aß-coef =–15.6, p value = 0.01) in the breast milk.	II-1	Limited sample-size; Heterogeneous schedule for milk sampling; Food intake was assessed for the 24 h preceding the sampling.

Alur, et al (2022) [17]	Narrative review	Not specified	Sexual dimorphism	Not specified	The milk from mothers of males had a higher energy, fat, and carbohydrate content than the milk from mothers of females	Not applicable	Not specified
Ryoo, et al. (2022) [43]	Cross-sectional, at 2 weeks postpartum	159 mothers of term infants	Maternal dietary intake<br>Others (stress, sleep quality, spousal support)	Miris†	The %energy from carbohydrates (β = 0.86, $p < 0.01$) and %energy from fat (β = 0.77, $p < 0.05$) intake were positively correlated with the breast milk energy content<br>No association was found between postpartum stress, sleep quality, and spousal support and the breast milk macronutrient content	II-1	Convenience sampling<br>Participants with the same daily diet at equal hours<br>Only 'transitional breast milk' studied
Adhikari, et al. (2022) [24]	Systematic review of 50 studies	Not specified	Maternal nutritional status<br>Maternal dietary intake	Various	Maternal nutritional status was positively associated with the fat content and negatively associated with protein content in milk<br>Neither nutrient intake nor nutritional status were associated with carbohydrate content	Not applicable	Large variation in assessment methods for maternal dietary intake, maternal nutritional status and HM composition
Koutsiafti, et al. (2021) [37]	Prospective	50 mothers of preterm and full-term infants	Socioeconomic status	Milkoscan 4000 device	The milk from mothers working in the private sector or that were self-employed had a significantly higher protein and fat content than the milk from mothers working in the public sector or that were unemployed ($p < 0.01$).	II-1	Limited sample size
Thakur, et al. (2021) [48]	Prospective, during the first 4 weeks postpartum	60 mothers delivering before or at 32 weeks of gestation	Lactation stage<br>Gestational age<br>Parity<br>Mode of delivery<br>Pre-pregnancy BMI	Miris†	During the first 4 weeks of lactation, the true protein content decreased, fat and energy content increased, and carbohydrate content remained stable<br>Macronutrient content was independent of analyzed maternal and neonatal factors	II-1	HM samples were collected in the morning rather than being pooled for 24 h

			Previous breastfeeding<br>Birth weight<br>Birth centile<br>Doppler flows				
Samuel, et al. (2020) [38]	Narrative review	Not specified	Maternal, infant, and methodological factors that affect breast milk composition	Not acknowledged	No consistent evidence of the effect of socioeconomic status on breast milk composition was found except that lower socioeconomic status was associated with lower n-3 long-chain polyunsaturated fatty acid content.	Not applicable	Not acknowledged
Leghi, et al. (2020) [25]	Systematic review of 31 studies, 9 of which included in the meta-analysis	5078 lactating mothers included in the review, and 872 in the meta-analysis	Measures of maternal overweight and obesity (anthropometry, BIS, and DXA, and %IBW)	Various	Maternal BMI and/or fat mass was positively correlated with breast milk fat content<br>The milk from overweight/ obese mothers had a higher lactose content in the colostrum and a lower fat content in the transition milk, than the milk from normal weight mothers	Not applicable	Considerable variability of results between studies<br>Low quality of many studies
Italianer, et al. (2020) [49]	Systematic review of 83 studies	200 mothers of term infants	Circadian rhythmicity	Various, but not specified	Significant circadian variation in total fat, triacylglycerol, and cholesterol content in the milk	Not applicable	The timing of the acrophase may be inaccurate due to differences in daylight and darkness in different countries, as well as seasonal fluctuations.

Galante, et al (2020) [18]	Narrative review	Not specified	Sexual dimorphism	Not specified	There is limited and conflicting evidence for sex-specificity in HM composition.	Not applicable	Not acknowledged
Hosseini, et al. (2020) [55]	Cross-sectional	119 mothers	Sexual dimorphism	Ultrasonic milk analyzer (Lactoscan MCC; Milk-otronic Company, Nova Zagora, Bulgaria)	The milk from mothers of males had a significantly lower fat content than the milk from mothers of females	II-1	Most of the participants were from East Azerbaijan<br>Small sample size<br>Only one HM sample collected per mother, no dietary data from mothers, and limited evaluated factors
Sahin, et al (2020) [47]	Prospective, during the first 4 weeks postpartum	60 mothers (39 of term and 21 of preterm infants)	Lactation stage<br>Mode of delivery	Miris†	Over time: protein content decreased in preterm and term groups ($p<0.001$, $p<0.001$, respectively); fat and carbohydrate content of preterm and term milk increased (for fat = ($p= 0.056$, $p<0.001$, respectively; for carbohydrates; $p< 0.001$, $p<0.001$, respectively); energy content increased in term milk but not in preterm milk ($p<0.001$, $p=0.026$, respectively)<br>The milk from mothers who delivered by cesarean section had a significantly higher protein content than the milk from mothers who delivered by vaginal delivery (1.794–0.848; 1.543–0.514g/dL respectively; $p=0.021$).	II-1	BMI of mothers was not calculated and taken into consideration<br>The total milk volume produced by each mother was not analyzed
Notarbartolo di Villarosa	Systematic review of 14 studies	Not specified	Maternal nutrition status<br>Hypertension	Various	The milk from overweight mothers had a higher fat and energy content		Not acknowledged

do Amaral, et al. (2019) [4]			Diabetes mellitus		The colostrum and mature milk from mothers with hypertension had higher levels of total protein than the milk from normotensive mothers<br>The milk from mothers with diabetes mellitus had a lower lactose and fat content than the milk from euglycemic mothers.		
Fischer Fumeaux, et al. (2019) [46]	Prospective, during 4 and 2 months postpartum for preterm and term deliveries, respectively	61 mothers (34 of term and 27 of preterm infants)	Lactation stage	Miris†	The protein content of both preterm and term milk decreased from birth to four months postpartum, with no significant difference between the two groups at any point during the postpartum period. However, preterm milk had a lower protein content at term equivalent age<br>Lactose content remained stable over time<br>Fat and energy content of preterm milk was higher in the first two weeks of lactation, whereas term milk had a higher fat and energy content later during lactation (three to eight weeks)<br>Male sex was associated with a higher fat and energy content in preterm and term milk	II-1	Small sample size in each group<br>One time breast milk collection<br>Dubious reliability of HM analyzer
Shapira, et al. (2019) [15]	Prospective, at 14 days postpartum	62 mothers of term infants	Gestational diabetes mellitus	Miris†	The milk from mothers with GDM was associated with a higher fat and energy content in mature milk (p=0.07) than the milk from euglycemic mothers (p <0.02)	II-1	HbA1C was not routinely measured
Burianova, et al. (2019) [42]	Prospective, during 6 weeks postpartum	192 mothers of preterm infants	Maternal BMI<br>Lactation stage<br>Parity<br>Mode of delivery<br>Smoking habits	Miris†	The milk from mothers with pre-pregnancy BMI ≥ 30 km/m^2 had a higher fat and energy content than the milk from mothers with BMI < 30 km/m^2<br>In normal-weight and overweight mothers, the mature milk fat content decreased overtime, whereas in obese mothers it did not vary (statistically significant interaction of pre-pregnancy BMI with time, p = 0.030)<br>Protein content decreased by three weeks postpartum and then remained stable until the end of the 6th week<br>Carbohydrate content increased to a stable level by the end of the 3rd week		The daily milk volume was not measured

					A higher protein content (p=0.001) and lower carbohydrate content (on average by 0.26g/dL, p=0.003) was found in the milk from primiparous compared to multiparous mothers<br>Parity was positively correlated with carbohydrate content in colostrum<br>Vaginal birth was positively associated with colostrum carbohydrate content (p=0.021)<br>Mature milk from smokers had a lower fat content than the milk from non-smokers (p = 0.026)		
Mimouni, et al. (2017) [12]	Systematic review of 23 studies, including 13 with meta-analysis of preterm milk, during 12 weeks postpartum	Not specified	Lactation stage	Not specified	There was a progressive decrease in protein content and a progressive increase in milk fat content over time<br>There was an initial increase in energy and carbohydrate content and then a steady state was reached	Not applicable	Not acknowledged
Dritsakou, et al. (2017) [39]	Prospective, on the 3rd, 7th and 30th day of lactation	305 mothers of preterm and term infants	Maternal age<br>Birth weight<br>Gestational age<br>Diet-controlled gestational diabetes<br>Postpartum maternal body fat	Miris†	Maternal age was positively correlated with fat content in colostrum, transitional and mature milk<br>Birth weight was negatively associated with fat and energy content in colostrum; with fat, carbohydrates and energy content in transitional milk; and with content of macronutrients and energy in mature milk<br>Gestational age was negatively associated with fat, carbohydrate and energy content in colostrum; with fat and energy content in transitional milk; and with content of macronutrients and energy in mature milk<br>Maternal body fat was positively associated with milk fat content	II-1	Number of milk samples from mothers with preterm birth was higher than samples from mothers with term birth<br>Colostrum was considered as the milk produced until

							the 5th day of lactation<br>No information was provided on the weight gain during pregnancy or pre-pregnancy BMI
Argov-Argaman, et al. (2016) [40]	Prospective, at 3, 7 and 14 days postpartum	49 mothers	al age	Creamatocrit method for fat and gas chromatography for fatty acids content	The transitional milk from younger mothers had a lower total fat content and a higher n-6 FA, EPA and ARA content	II-1	Not acknowledged
Hahn, et al. (2016) [52]	Cross-sectional	418 mothers	Sexual dimorphism<br>Birth height<br>Delivery mode<br>Postpartum age	Miris†	The milk from mothers of females had a higher carbohydrate (OR=0.56, p=0.012) and energy (OR=0.33, p=0.017) content<br>Birth height was positively associated with milk fat content (OR=0.84, p=0.004) and energy content (OR=0.74, p<0.001)<br>The milk from mothers who had a cesarean section (OR=2.47, p<0.001) was associated with a higher fat content than the milk from mothers with vaginal delivery<br>The milk from mothers who had vaginal delivery was associated with a higher carbohydrate content (OR=0.50, p=0.005) than the milk from mothers with cesarean section<br>Postpartum age was positively associated with the milk protein (OR=0.89, p<0.001) and energy content (OR=0.95, p=0.003)<br>Female infant (OR=0.36, p=0.029), birth height (OR=0.73, p=0.001), and postpartum age (OR=0.95, p=0.005) were independent risk factors for a higher milk energy content	II-1	Maternal diet not controlled<br>There were differences in sampling times<br>Data of maternal anthropometry were not collected

Gidrewicz, et al. (2014) [51]	Systematic review and meta-analysis of 41 studies	3142 mothers of preterm and term infants	Gestational age	Various	True protein content was higher in preterm milk, with maximum mean differences up to 35% in the first 3 days postpartum compared to term milk<br>Lactose content was significantly lower in preterm milk compared to term milk<br>Fat content considered not differing statistically (all p-values > 0.001) between preterm and term milk at any point of lactation	Not applicable	Low availability of results from individual studies<br>Small sample sizes
Dizdar, et al. (2014) [53]	Prospective, on the 2nd postpartum day	24 mothers of term infants	Mode of delivery	Mirist	Vaginal delivery was associated with a higher milk protein content in colostrum than in cesarean section	II-1	Not acknowledged
Quinn, et al. (2013) [57]	Cross-sectional	103 mothers	Sexual dimorphism	Micro-Rose Gottlieb for fat; total nitrogen on an Elmer Perkins CHN analyzer and converted to total protein using standard formulas; Phenol-Sulfuric Acid for sugars; and standard formulas for energy content	No differences in milk content were found regarding infant sex	II-1	Not acknowledged

Bachour, et al. (2012) [45]	Cross-sectional	66 mothers	Lactation stage<br>Maternal BMI<br>Parity<br>Smoking habits<br>Maternal age	Gravimetric method for fat and Bradford method for protein	In mature milk, the protein content decreased by 20% (p=0.015) and fat content increased by 25% (p=0.034), in comparison with transitional milk.<br>Overweight mothers' milk had 14.9% less protein content than the milk from normal weight mothers (p=0.044) and 18.7% less than the milk from obese mothers (p=0.033)<br>Fat content increased in parallel with an increase in parity number up to 3 (F=11.87, p<0.05)<br>In smokers there was a 26% decrease in fat content (t test, p=0.015) and a 12% decrease in protein content (t test, p=0.048), compared with non-smokers	II-1	Not acknowledged
Bauer, et al. (2011) [6]	Prospective,during the first 8 weeks postpartum	113 mothers delivering between 23-33 weeks of gestation and 10 mothers delivering at term	Macronutrients and energy content	Ballistic bomb calorimeter for energy<br>Lowry method for protein<br>Orcinol assay for carbohydrate<br>Creamatocrit for fat	Carbohydrate, fat and energy content was significantly higher in preterm than in term milk.<br>Protein content was significantly higher in the milk from mothers delivering before 28 weeks of gestation than either in the milk from mothers delivering between 32-33 weeks or after 37 weeks of gestation	II-1	Minor concerns with the methods used for HM analysis
Campbell-Yeo, et al (2010) [50]	Clinical trial, at 7 and 14 days postpartum	46 mothers	Galactagogues	Kjeldahl Method used for total nitrogen; Roese-Gottlib method for fat; standardized	The milk from mothers taking domperidone had a lower protein and higher carbohydrate and calcium content compared with the milk from mothers taking placebo.	I	Not acknowledged

				methods for carbohydrate and energy content			
Powe, et al. (2009) [56]	Cross-sectional	25 mothers	Sexual dimorphism	Kjeldahl procedure was used for total nitrogen; Modified method from the Association of Official Analytical Chemists for fat; Total carbohydrates and total energy was estimated	The milk from mothers of males had a higher energy content than the milk from mothers of females	II-1	Small sample size<br>Some data was collected by self-report

Abbreviations: AGA, appropriate for gestational age; ALA, alpha-linolenic acid; ARA, arachidonic acid; BMI, body mass index; BIS, bioelectrical impedance spectroscopy; DHA, docosahexaenoic acid; DXA, dual-energy X-ray absorptiometry; EPA, eicosapentaenoic acid; GDM, gestational diabetes mellitus; HM, human milk; IUGR, intrauterine growth restriction; PUFA, polyunsaturated fatty acids; SGA, small for gestational age; %IBW, pre-pregnancy percent ideal body weight. †Miris - Miris Human Milk Analyzer (Miris AB, Uppsala, Sweden)

References

1. Cao, Y.; Jiang, S.; Sun, J.; Hei, M.; Wang, L.; Zhang, H.; Ma, X.; Wu, H.; Li, X.; Sun, H.; et al. Assessment of neonatal intensive care unit practices, morbidity, and mortality among very preterm infants in China. *JAMA Netw. Open* **2021**, *4*, e2118904. https://doi.org/10.1001/jamanetworkopen.2021.18904.
2. Alanazi, M.; Altawili, M.A.; Khayyal, A.I.; Alahmari, A.S.; Alhakami, A.A.; Alshehri, A.M.A. Impact of early nutrition interventions on the growth and development of preterm infants: A narrative review. *Cureus* **2024**, *16*, e54888. https://doi.org/10.7759/cureus.54888.
3. Ruys, C.A.; van de Lagemaat, M.; Rotteveel, J.; Finken, M.J.J.; Lafeber, H.N. Improving long-term health outcomes of preterm infants: How to implement the findings of nutritional intervention studies into daily clinical practice. *Eur. J. Pediatr.* **2021**, *180*, 1665–1673. https://doi.org/10.1007/s00431-021-03950-2.
4. Notarbartolo di Villarosa do Amaral, Y.; Marano Rocha, D.; Lopes da Silva, L.M.; Valente Mendes Soares, F.; Lopes Moreira, M.E. Do maternal morbidities change the nutritional composition of human milk?—A systematic review. *Cienc. Saude Coletiva* **2019**, *24*, 2491–2498. https://doi.org/10.1590/1413-81232018247.18972017.
5. Petersohn, I.; Hellinga, A.H.; van Lee, L.; Keukens, N.; Bont, L.; Hettinga, K.A.; Feskens, E.J.M.; Brouwer-Brolsma, E.M. Maternal diet and human milk composition: An updated systematic review. *Front. Nutr.* **2024**, *10*, 1320560. https://doi.org/10.3389/fnut.2023.1320560.
6. Bauer, J.; Gerss, J. Longitudinal analysis of macronutrients and minerals in human milk produced by mothers of preterm infants. *Clin. Nutr.* **2011**, *30*, 215–220. https://doi.org/10.1016/j.clnu.2010.08.003.
7. Borràs-Novell, C.; Herranz Barbero, A.; Balcells Esponera, C.; López-Abad, M.; Aldecoa Bilbao, V.; Izquierdo Renau, M.; Iglesias Platas, I. Influence of maternal and perinatal factors on macronutrient content of very preterm human milk during the first weeks after birth. *J. Perinatol.* **2022**, *43*, 52–59. https://doi.org/10.1038/s41372-022-01475-6.
8. Favara, G.; Maugeri, A.; Barchitta, M.; Lanza, E.; Magnano San Lio, R.; Agodi, A. Maternal lifestyle factors affecting breast milk composition and infant health: A systematic review. *Nutrients* **2024**, *17*, 62. https://doi.org/10.3390/nu17010062.
9. Hashemi Javaheri, F.S.; Karbin, K.; Senobari, M.A.; Hakim, H.G.; Hashemi, M. The association between maternal body mass index and breast milk composition: A systematic review. *Nutr. Rev.* **2025**, *83*, 83–111. https://doi.org/10.1093/nutrit/nuad174.
10. Voerman, E.; Santos, S.; Patro Golab, B.; Amiano, P.; Ballester, F.; Barros, H.; Bergström, A.; Charles, M.A.; Chatzi, L.; Chevrier, C.; et al. Maternal body mass index, gestational weight gain, and the risk of overweight and obesity across childhood: An individual participant data meta-analysis. *PLoS Med.* **2019**, *16*, e1002744. https://doi.org/10.1371/journal.pmed.1002744.
11. Ellsworth, L.; Perng, W.; Harman, E.; Das, A.; Pennathur, S.; Gregg, B. Impact of maternal overweight and obesity on milk composition and infant growth. *Matern. Child. Nutr.* **2020**, *16*, e12979. https://doi.org/10.1111/mcn.12979.
12. Mimouni, F.B.; Lubetzky, R.; Yochpaz, S.; Mandel, D. Preterm Human Milk Macronutrient and Energy Composition—A systematic review and meta-analysis. *Clin. Perinatol.* **2017**, *44*, 165–172. https://doi.org/10.1016/j.clp.2016.11.010.
13. Correia, L.; Cardoso, M.; Papoila, A.L.; Alves, M.; Virella, D.; Ramalho, R.; Pereira, P.; Macedo, I.; Tomé, T.; Cohen, Á.; et al. Does fetal growth adequacy affect the nutritional composition of mothers' milk? A historical cohort study. *Am. J. Perinatol.* **2021**, *40*, 163–171. https://doi.org/10.1055/s-0041-1727278.

14. Arenas, G.; Barrera, M.J.; Contreras-Duarte, S. The impact of maternal chronic inflammatory conditions on breast milk composition: Possible influence on offspring metabolic programming. *Nutrients* **2025**, *17*, 387. https://doi.org/10.3390/nu17030387.
15. Shapira, D.; Mandel, D.; Mimouni, F.B.; Moran-Lev, H.; Marom, R.; Mangel, L.; Lubetzky, R. The effect of gestational diabetes mellitus on human milk macronutrients content. *J. Perinatol.* **2019**, *39*, 820–823. https://doi.org/10.1038/s41372-019-0362-5.
16. Viswanathan, S.; Thoene, M.; Alja'nini, Z.; Alur, P.; McNelis, K. Body composition in preterm infants: Current insights and emerging perspectives. *Children* **2025**, *12*, 53. https://doi.org/10.3390/children12010053.
17. Alur, P.; Ramarao, S. Sex differences in preterm nutrition and growth: The evidence from human milk associated studies. *J. Perinatol.* **2022**, *42*, 987–992. https://doi.org/10.1038/s41372-022-01354-0.
18. Galante, L.; Milan, A.M.; Reynolds, C.M.; Cameron-Smith, D.; Vickers, M.H.; Pundir, S. Sex-specific human milk composition: The role of infant sex in determining early life nutrition. *Nutrients* **2018**, *10*, 1194. https://doi.org/10.3390/nu10091194.
19. Arslanoglu, S.; Boquien, C.-Y.; King, C.; Lamireau, D.; Tonetto, P.; Barnett, D.; Bertino, E.; Gaya, A.; Gebauer, C.; Grovslien, A.; et al. Fortification of human milk for preterm infants: Update and recommendations of the European Milk Bank Association (EMBA) Working Group on Human Milk Fortification. *Front. Pediatr.* **2019**, *7*, 76. https://doi.org/10.3389/fped.2019.00076.
20. Leghi, G.E.; Middleton, P.F.; Netting, M.J.; Wlodek, M.E.; Geddes, D.T.; Muhlhausler, B.S. A systematic review of collection and analysis of human milk for macronutrient composition. *J. Nutr.* **2020**, *150*, 1652–1670. https://doi.org/10.1093/jn/nxaa059.
21. Czosnykowska-Łukacka, M.; Królak-Olejnik, B.; Orczyk-Pawiłowicz, M. Breast milk macronutrient components in prolonged lactation. *Nutrients* **2018**, *10*, 1893. https://doi.org/10.3390/nu10121893.
22. Billard, H.; Simon, L.; Desnots, E.; Sochard, A.; Boscher, C.; Riaublanc, A.; Alexandre-Gouabau, M.-C.; Boquien, C.-Y. Calibration adjustment of the mid-infrared analyzer for an accurate determination of the macronutrient composition of human milk. *J. Hum. Lact.* **2015**, *32*, NP19–NP27. https://doi.org/10.1177/0890334415588513.
23. Giuffrida, F.; Austin, S.; Cuany, D.; Sanchez-Bridge, B.; Longet, K.; Bertschy, E.; Sauser, J.; Thakkar, S.K.; Lee, L.Y.; Affolter, M. Comparison of macronutrient content in human milk measured by mid-infrared human milk analyzer and reference methods. *J. Perinatol.* **2018**, *39*, 497–503. https://doi.org/10.1038/s41372-018-0291-8.
24. Adhikari, S.; Kudla, U.; Nyakayiru, J.; Brouwer-Brolsma, E.M. Maternal dietary intake, nutritional status and macronutrient composition of human breast milk: Systematic review. *Br. J. Nutr.* **2022**, *127*, 1796–1820. https://doi.org/10.1017/s0007114521002786.
25. Leghi, G.E.; Netting, M.J.; Middleton, P.F.; Wlodek, M.E.; Geddes, D.T.; Muhlhausler, B.S. The impact of maternal obesity on human milk macronutrient composition: A systematic review and meta-analysis. *Nutrients* **2020**, *12*, 934. https://doi.org/10.3390/nu12040934.
26. Embleton, N.D.M.; Moltu, S.J.; Lapillonne, A.; van den Akker, C.H.; Carnielli, V.; Fusch, C.; Gerasimidis, K.; van Goudoever, J.B.; Haiden, N.M.; Iacobelli, S.; et al. Enteral nutrition in preterm infants (2022): A position paper from the ESPGHAN Committee on Nutrition and Invited Experts. *J. Pediatr. Gastroenterol Nutr.* **2023**, *76*, 248–268.

27. World Health Organization. WHO Recommendations for Care of the Preterm or Low-Birth-Weight Infant. 2022. Available online: https://www.who.int/news-room/fact sheets/detail/preterm-birth (accessed on 16 June 2025).
28. Brown, J.V.E.; Lin, L.; Embleton, N.D.; Harding, J.E.; McGuire, W. Multi-nutrient fortification of human milk for preterm infants. *Cochrane Database Syst. Rev.* **2020**, *6*, CD000343. https://doi.org/10.1002/14651858.cd000343.pub4.
29. Arslanoglu, S.; Moro, G.E.; Ziegler, E.E. Adjustable fortification of human milk fed to preterm infants: Does it make a difference? *J. Perinatol.* **2006**, *26*, 614–621. https://doi.org/10.1038/sj.jp.7211571.
30. Cardoso, M.; Virella, D.; Papoila, A.L.; Alves, M.; Macedo, I.; Silva, D.E.; Pereira-Da-Silva, L. Individualized fortification based on measured macronutrient content of human milk improves growth and body composition in infants born less than 33 weeks: A mixed-cohort study. *Nutrients* **2023**, *15*, 1533. https://doi.org/10.3390/nu15061533.
31. World Health Organization. Social Determinants of Health. Available online: https://www.who.int/health-topics/social-determinants-of-health#tab=tab_1 (accessed on 23 July 2025).
32. Miris. About Us. Available online: https://www.mirissolutions.com/about-us (accessed on 20 July 2025).
33. Moher, D.; Liberati, A.; Tetzlaff, J.; Altman, D.G.; PRISMA Group. Preferred reporting items for systematic reviews and meta-analyses: The PRISMA statement. *PLoS Med.* **2009**, *6*, e1000097. https://doi.org/10.1371/journal.pmed.1000097.
34. Burns, P.B.; Rohrich, R.J.; Chung, K.C. The levels of evidence and their role in evidence-based medicine. *Plast. Reconstr. Surg.* **2011**, *128*, 305–310. https://doi.org/10.1097/PRS.0b013e318219c171.
35. Chathyushya, K.B.; Hemalatha, R.; Ananthan, R.; Babu, J.J.; Devraj, J.P.; Banjara, S.K.; Alimelu, M.; Pradeep, R.K.; Nitasha, B.; Shiva, P.M. Macronutrient composition of term and preterm human milk of different socio economic groups. *Prostaglandins Leukot. Essent. Fat. Acids* **2023**, *192*, 102571. https://doi.org/102571. 10.1016/j.plefa.2023.102571.
36. Bottin, J.H.; Eussen, S.R.B.M.; Igbinijesu, A.J.; Mank, M.; Koyembi, J.J.; Nyasenu, Y.T.; Ngaya, G.; Mad-Bondo, D.; Kongoma, J.B.; Stahl, B.; et al. Food insecurity and maternal diet influence human milk composition between the infant's birth and 6 months after birth in Central-Africa. *Nutrients* **2022**, *14*, 4015. https://doi.org/10.3390/nu14194015.
37. Koutsiafti, P.; Soultani, G.; Kechagias, S.; Grivea, I.; Malissiova, E. Macronutrient composition of breast milk for full term and premature infants: Correlation to nutritional and socioeconomic factors. *Int. J. Caring Sci.* **2021**, *14*, 608–616.
38. Samuel, T.M.; Zhou, Q.; Giuffrida, F.; Munblit, D.; Verhasselt, V.; Thakkar, S.K. Nutritional and non-nutritional composition of human milk is modulated by maternal, infant, and methodological factors. *Front. Nutr*. **2020**, *7*, 576133. https://doi.org/10.3389/fnut.2020.576133.
39. Dritsakou, K.; Liosis, G.; Valsami, G.; Polychronopoulos, E.; Skouroliakou, M. The impact of maternal- and neonatal-associated factors on human milk's macronutrients and energy. *J. Matern. Fetal Neonatal Med.* **2016**, *30*, 1302–1308. https://doi.org/10.1080/14767058.2016.1212329.
40. Argov-Argaman, N.; Mandel, D.; Lubetzky, R.; Hausman Kedem, M.; Cohen, B.-H.; Berkovitz, Z.; Reifen, R. Human Milk Fatty acids composition is affected by maternal age. *J. Matern. Neonatal Med.* **2016**, *30*, 34–37. https://doi.org/10.3109/14767058.2016.1140142.

41. Bravi, F.; Wiens, F.; Decarli, A.; Dal Pont, A.; Agostoni, C.; Ferraroni, M. Impact of maternal nutrition on breast-milk composition: A systematic review. *Am. J. Clin. Nutr.* **2016**, *104*, 646–662. https://doi.org/10.3945/ajcn.115.120881.
42. Burianova, I.; Bronsky, J.; Pavlikova, M.; Janota, J.; Maly, J. Maternal body mass index, parity and smoking are associated with human milk macronutrient content after preterm delivery. *Early Hum. Dev.* **2019**, *137*, 104832. https://doi.org/10.1016/j.earlhumdev.2019.104832.
43. Ryoo, C.J.; Kang, N.M. Maternal factors affecting the macronutrient composition of transitional human milk. *Int. J. Environ. Res. Public. Health* **2022**, *19*, 3308. https://doi.org/10.3390/ijerph19063308.
44. Marano, D.; Melo, R.X.; da Silva, D.A.; Vilarim, M.M.; Moreira, M.E.L. Nutritional composition of human milk and its association with maternal and perinatal factors. *Rev. Paul. Pediatr.* **2023**, *42*, e2023001. https://doi.org/10.1590/1984-0462/2024/42/2023001.
45. Bachour, P.; Yafawi, R.; Jaber, F.; Choueiri, E.; Abdel-Razzak, Z. Effects of smoking, mother's age, body mass index, and parity number on lipid, protein, and secretory immunoglobulin a concentration of human milk. *Breastfeed. Med.* **2012**, *7*, 179–188. https://doi.org/10.1089/bfm.2011.0038.
46. Fischer Fumeaux, C.J.; Garcia-Rodenas, C.L.; De Castro, C.A.; Courtet-Compondu, M.-C.; Thakkar, S.K.; Beauport, L.; Tolsa, J.-F.; Affolter, M. Longitudinal analysis of macronutrient composition in preterm and term human milk: A prospective cohort study. *Nutrients* **2019**, *11*, 1525. https://doi.org/10.3390/nu11071525.
47. Sahin, S.; Ozdemir, T.; Katipoglu, N.; Akcan, A.B.; Kaynak Turkmen, M. Comparison of changes in breast milk macronutrient content during the first month in preterm and term infants. *Breastfeed. Med.* **2020**, *15*, 56–62. https://doi.org/10.1089/bfm.2019.0141.
48. Thakur, A.; Kler, N.; Garg, P.; Gandhi, P.; Srivastava, S. Macronutrient analysis of human milk and factors associated with its composition in mothers of preterm infants ≤ 32 weeks. *Eur. J. Pediatr.* **2021**, *180*, 3527–3534. https://doi.org/10.1007/s00431-021-04158-0.
49. Italianer, M.F.; Naninck, E.F.G.; Roelants, J.A.; Van Der Horst, G.T.; Reiss, I.K.M.; van Goudoever, J.B.; Joosten, K.F.M.; Chaves, I.; Vermeulen, M.J. Circadian variation in human milk composition, a systematic review. *Nutrients* **2020**, *12*, 2328. https://doi.org/10.3390/nu12082328.
50. Campbell-Yeo, M.L.; Allen, A.C.; Joseph, K.S.; Ledwidge, J.M.; Caddell, K.; Allen, V.M.; Dooley, K.C. Effect of domperidone on the composition of preterm human breast milk. *Pediatrics* **2010**, *125*, e107–e114. https://doi.org/10.1542/peds.2008-3441.
51. Gidrewicz, D.A.; Fenton, T.R. A systematic review and meta-analysis of the nutrient content of preterm and term breast milk. *BMC Pediatr.* **2014**, *14*, 216. https://doi.org/10.1186/1471-2431-14-216.
52. Hahn, W.-H.; Song, J.-H.; Song, S.; Kang, N.M. Do gender and birth height of infant affect calorie of human milk? An association study between human milk macronutrient and various birth factors. *J. Matern. Fetal Neonatal Med.* **2016**, *30*, 1608–1612. https://doi.org/10.1080/14767058.2016.1219989.
53. Dizdar, E.A.; Sari, F.N.; Degirmencioglu, H.; Canpolat, F.E.; Oguz, S.S.; Uras, N.; Dilmen, U. Effect of mode of delivery on macronutrient content of breast milk. *J. Matern. Fetal Neonatal Med.* **2013**, *27*, 1099–1102. https://doi.org/10.3109/14767058.2013.850486.
54. Mammaro, A.; Carrara, S.; Cavaliere, A.; Ermito, S.; Dinatale, A.; Pappalardo, E.M.; Militello, M.; Pedata, R. Hypertensive disorders of pregnancy. *J. Prenat. Med.* **2009**, *3*, 1–5.

55. Hosseini, M.; Valizadeh, E.; Hosseini, N.; Khatibshahidi, S.; Raeisi, S. The Role of Infant Sex on Human Milk Composition. *Breastfeed. Med.* **2020**, *15*, 341–346. https://doi.org/10.1089/bfm.2019.0205.
56. Powe, C.E.; Knott, C.D.; Conklin-Brittain, N. Infant sex predicts breast milk energy content. *Am. J. Hum. Biol.* **2009**, *22*, 50–54. https://doi.org/10.1002/ajhb.20941.
57. Quinn, E.A. No evidence for sex biases in milk macronutrients, energy, or breastfeeding frequency in a sample of Filipino mothers. *Am. J. Phys. Anthropol.* **2013**, *152*, 209–216. https://doi.org/10.1002/ajpa.22346.
58. Khelouf, N.; Haoud, K.; Meziani, S.; Fizir, M.; Ghomari, F.N.; Khaled, M.B.; Kadi, N. Effect of infant's gender and lactation period on biochemical and energy breast milk composition of lactating mothers from Algeria. *J. Food Compos. Anal.* **2023**, *115*, 104889. https://doi.org/10.1016/j.jfca.2022.104889.
